# Development of a Transplantable GFP+ B-Cell Lymphoma Tumor Cell Line From MHC-Defined Miniature Swine: Potential for a Large Animal Tumor Model

**DOI:** 10.3389/fonc.2019.00209

**Published:** 2019-04-02

**Authors:** Marian Schenk, Abraham J. Matar, Isabel Hanekamp, Robert J. Hawley, Christene A. Huang, Raimon Duran-Struuck

**Affiliations:** ^1^Center for Transplantation Sciences, Massachusetts General Hospital, Charlestown, MA, United States; ^2^Department of Surgery, Emory University, Atlanta, GA, United States; ^3^Department of Surgery, University of Colorado-Denver, Denver, CO, United States; ^4^Department of Pathobiology, University of Pennsylvania School of Veterinary Medicine, Philadelphia, PA, United States

**Keywords:** MHC defined miniature swine, GFP+ tumor cell line, transplantable swine tumors, swine lymphoma tumor cell line, large animal tumor model

## Abstract

The lack of a reliable and reproducible large animal tumor model for the study of hemolymphatic malignancies limits the ability to explore the underlying pathophysiology and testing of novel therapies. The goal of this study was to develop an aggressive, trackable swine tumor cell line in mice for adoptive transfer into MHC matched swine. Two tumor cell lines, post-transplant lymphoproliferative disease (PTLD) 13271 and chronic myelogenous leukemia (CML) 14736, were previously established from the Massachusetts General Hospital (MGH) miniature swine herd. PTLD 13271 is a swine B-cell lymphoma line originating from an animal that developed PTLD following hematopoietic cell transplantation (HCT), while CML 14736 was generated from a swine that spontaneously developed CML. In order to select for aggressive tumor variants, both lines were passage into NOD/SCID IL-2 receptor γ^−/−^ (NSG) mice. Tumor induced mortality in mice injected with CML14736 was 68% while 100% of mice injected with PTLD 13271 succumbed to PTLD by day 70. Based on aggressiveness, PTLD 13271 was selected for further development and re-passage into NSG mice resulting in increased tumor burden and metastasis. Transduction of the PTLD 13271 cell line with a green fluorescent protein (GFP)-expressing lentivirus facilitated tumor tracking when re-passaged in mice. Utilizing a tolerance induction strategy, GFP+ tumors were injected into an MHC matched miniature swine and successfully followed via flow cytometry for 48 h in circulation, although tumor engraftment was not observed. In summary, we report the development of an aggressive GFP+B-cell lymphoma cell line which has the potential for facilitating development of a large animal tumor model.

## Introduction

Although several murine models of hemolymphatic malignancies exist, they are often limited in their reproducibility when translated to clinical practice (1, 2). A pre-clinical large animal model would more closely mimic human disease, allowing for improved understanding of tumor biology and response to novel therapies. Compared to other large animals, miniature swine are an excellent modality for the study of hemolymphatic malignancies for various reasons including a similar size, anatomy, and physiology to humans, short time to sexual maturity, and the relative ease of breeding and handling (3). Specifically, the Massachusetts General Hospital (MGH) miniature swine herd is a clinically relevant large animal model based on its unique genetic characteristics (4). These swine have been characterized and fixed at the major histocompatibility complex (MHC) making them ideal for studies in transplantation.

Hemolymphatic malignancies from the MGH miniature swine herd have previously been harvested and maintained *in vitro* for the development of a large animal tumor model (5). Prior attempts at adoptive transfer of tumors into naïve MHC matched swine have not resulted in consistent tumorigenesis. Cho et al. reported subcutaneous (SQ), but not systemic, tumor growth in conditioned naïve MHC matched swine when transferring CML 14736, a chronic myelogenous leukemia (CML) tumor cell line (5). SQ tumor growth was only observed in animals receiving at least 300 cGY of total body irradiation (TBI), which ultimately led to unacceptable levels of toxicity. Another well-characterized tumor cell line developed in our laboratory is PTLD 13271, a B-cell lymphoma isolated from a haplotype AD swine that developed post-transplant lymphoproliferative disease (PTLD) following haploidentical hematopoietic cell transplantation (HCT). This tumor cell line has previously been shown to grow in NOD/SCID mice when injected intraperitoneally (IP) but has not yet been introduced into swine (5).

Based on these previous studies, our data suggested that transferring a primary tumor cell line into naïve swine conditioned with only irradiation was not sufficient to achieve consistent systemic tumor growth. Therefore, we hypothesized that selecting for aggressive tumor subclones through serial passage into mice combined with a “tolerance induction” strategy for tumor engraftment would lead to more reliable tumor growth. In this study, we passaged two established tumor cell lines (CML 14736 and PTLD 13271) through NSG mice (NOD/SCID IL2 γ^−/−^), which lack the IL2 receptor common gamma chain, generating aggressive tumor subclones for transfer into swine. Further, PTLD 13271 was successfully transduced with a green fluorescent protein (GFP)-expressing lentivirus to facilitate tumor tracking in both mice and swine. Here, we report on the development of aggressive, trackable swine tumor cell clones and our experience with serial transfer into MHC matched swine utilizing a tolerance induction approach.

## Materials and Methods

### Animals

NSG mice were used for these experiments (6). Swine used were from the unique inbred herd from the MGH previously described (4, 7). Animals used were haplotype swine leukocyte antigen AD (SLA^AD^), approximately 3 months of age. All animal care procedures as well as diagnostic and euthanasia procedures were approved by the MGH Subcommittee on Research Animal Care (SRAC).

### Tumor Inductions and Monitoring of NSG Mice

All mice received injections of 10 × 10^6^ tumor cells intravenously (IV) via the tail vein. Mice were observed daily for signs and symptoms of illness: weight loss/gain, fur integrity, activity, mobility, posture, abdominal distention, and abdominal growth. Once a certain degree of illness was noted as determined by MGH SRAC policy, animals were humanely euthanized and necropsy was performed.

### Antibodies and Flow Cytometry

Antibodies used for flow cytometry were: CD1 76-7-4, CD2-MSA-4, CD3 898H2-6-15, CD4 74-12-4, CD5 BBG-9G12, CD8 76-2-11, CD16 G7, CD172a 74-22-15, Class I 2.27.3a, Class II DR 40D, Class II DQ TH16, CD21 BB6-11C9, CD25 231.3B2, anti-mu heavy chain 5C9, and anti-k light-chain K139 3E1. Antibodies used for determination of mouse tumor origin were: CD2-MSA-4,12.2.2, H-2Dd 34-2-12, and Pan Pig. Flow cytometry was performed as previously described using a Becton Dickinson (BD) FACS Caliber (8). Winlist mode analysis software was used to analyze data.

### Immune Conditioning Regimens for Swine 20368 and 20984

Swine 20368 received a non-myeloablative preparatory regimen consisting of 200 cGY TBI, T-cell depletion with CD3 immunotoxin and a 45-day course of cyclosporine (CyA). Beginning on day −4 to −1, the animal received 50 μg/kg CD3 immunotoxin IV, twice daily. On day −2, the animal received 200 cGY TBI. Neoral cyclosporine was administered via a gastric tube twice daily at a dose of 15 mg/kg starting on day −1. Cyclosporine was continued for 60 days with a target range of 400–800 μg/ml. Swine 20984 received the same conditioning regimen as swine 20368 with two variations-1,000 cGY thymic irradiation (TI) was used instead of 200 cGY TBI and a slightly longer course of cyclosporine, 60 days instead of 45 days.

### Tumor Cell Preparation and Transfer Into Swine

Tumor cells were cultured in RPMI media and prepared by expansion in T175 flasks in an incubator at 37°C and 5% CO_2_. Prior to transfer *in vivo*, they were resuspended in PBS. Tumor cells were injected IV over a period of 1.5 h. Swine 20386 received three injections of cells. The first dose on day 0 consisted of 274 × 10^6^ cells/kg. The second dose of 300 × 10^6^ cells/kg was given on day 25. The third dose of 437 × 10^6^ cells/kg was given on day 53. Swine 20984 received only one injection of cells on day 0 of 385 × 10^6^ cells/kg.

### Monitoring Swine for Tumor Development

Animals were monitored daily for physical and laboratory signs of tumor development. Laboratory monitoring consisted of biweekly complete blood counts (CBC), chemistry, lactate dehydrogenase (LDH), and flow cytometry analysis. Swine 20984 underwent bone marrow biopsy and midline laparotomy for mesenteric lymph node analysis on day 21.

### Transduction of Murine Grown Swine PTLD 13271 With GFP Viral Vector

Tumor cells in log phase at a concentration of 1.8 × 10^6^ tumor cells/1.6 ml were added to retronectin coated plates along with two concentrations of pHAGE-GFP lentivirus. The plates were centrifuged at 600 g for 60 min. After spinoculation, cells were incubated at 37 C for 24 h prior to transfer to a T25 flask. The construct used was pHAGE-full EF1a-MCS-IZsGreen-W, which has been shown to successfully transduce swine cells. This lentivirus was made from the pHAGE-CMV-fullEF1a-IRES-ZsGreen plasmid and viruses were generated by five-plasmid co-transfection (HIV gag-pol, rev, tat, Env, and the vector plasmid) into 293T cells. The ENV plasmid encodes VSV-G.

### Data Analysis

Survival curves were evaluated using a Gehan-Breslow-Wilcoxon test. GraphPad Prism software was used for the figures and statistical analysis.

## Results

### Passage of PTLD 13271 in NSG Mice Results in 100% Tumor Engraftment and Mortality

**Figure 1:**
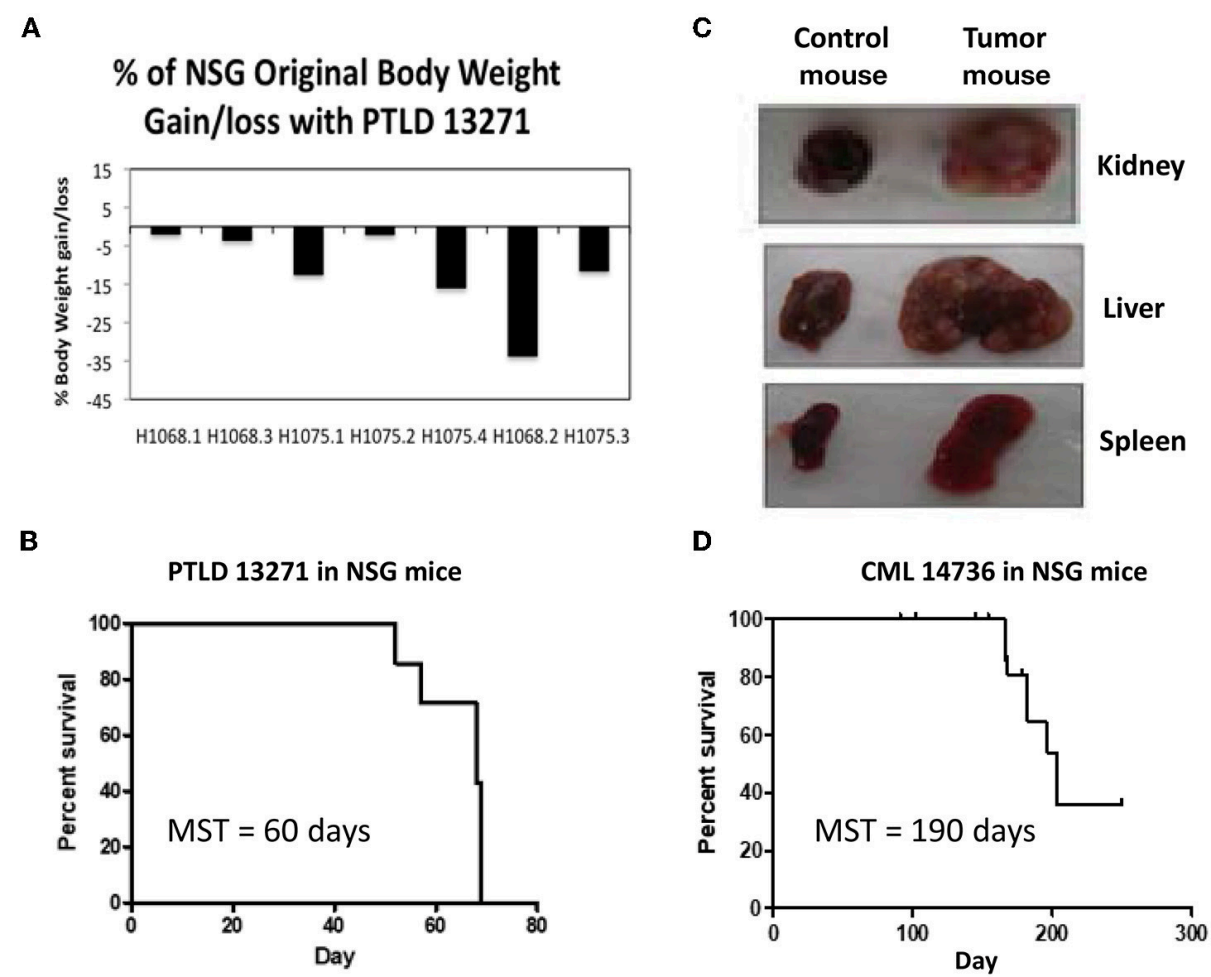
**(A)** The percent of original weight of NSG mice that was lost/gained after administration of PTLD tumor cells before death. 5/7 mice had a significant weight loss, while two stayed relatively the same. **(B)** Survival curve for the 7 NSG mice injected with PTLD 13271. Median survival time (MST) = 60 days. **(C)** Representative gross pathology from the first passage of PTLD 13271 into NSG demonstrates severe organomegaly compared to normal controls. **(D)** Survival curve for the 19 NSG mice injected with CML 14736. 13/19 mice (68%) developed swine CML with a MST = 190 days. Notch marks represent mice that died but did not have swine CML.

NSG mice, which are known to be devoid of NK cells in addition to T and B cells, were used for passage of swine tumor cell lines. All NSG mice (*n* = 7) were injected IV with 10 × 10^6^ PTLD 13271 cells. PTLD 13271 is a tumor cell line isolated from swine 13271, which developed an aggressive host type B-cell lymphoma (PTLD) following HCT. At day 50 post injection, mice began to show signs of tumor growth, including weight loss, lethargy, ruffled fur, and enlarged abdomens (Figure 1A). By day 70, all NSG mice had succumbed to PTLD (MST = 60 days) (Figure 1B). Necropsy revealed organomegaly and lymphadenopathy in all mice (Figure 1C).

### Passage of CML 14736 in NSG Mice Results in Delayed Tumor Engraftment and Inconsistent Mortality

We then tested the ability of a swine CML cell line to induce tumorigenesis in NSG mice. CML 14736 is a CML cell line isolated from an inbred haplotype DD swine, 14736, which spontaneously developed CML at the age of 2 years (9). Nineteen mice were each injected IV with 10 × 10^6^ CML 14736 cells. There were no signs of tumor growth until day 180. At day 180, 13 of 19 (68%) NSG mice began to show signs of tumor development, including weight loss, lethargy, ruffled fur, and enlarged abdomens. The median survival time (MST) was 190 days (Figure 1D).

### *In vivo* Harvested Tumors Retain Swine-Specific Immunophenotype

**Figure 2:**
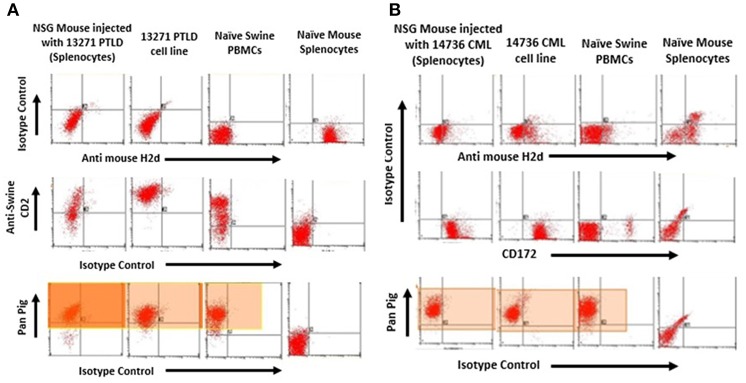
**(A)** Flow cytometry to confirm tumors from NSG injected with PTLD 13271 were of swine origin. Representative plot of one of the tumors harvested from the spleen of one of the injected mice was positive for the swine pan-pig (MHC-I) marker. These were negative for H2D^d^ mouse marker. **(B)** Tumors from NSG injected with CML 14736 were of swine origin. Representative flow cytometry of one of the tumors harvested from the spleen of one of the injected mice was positive for the swine pan-pig (MHC-I) marker (Highlighted region). These were negative for H2Dd mouse marker.

**Figure 3:**
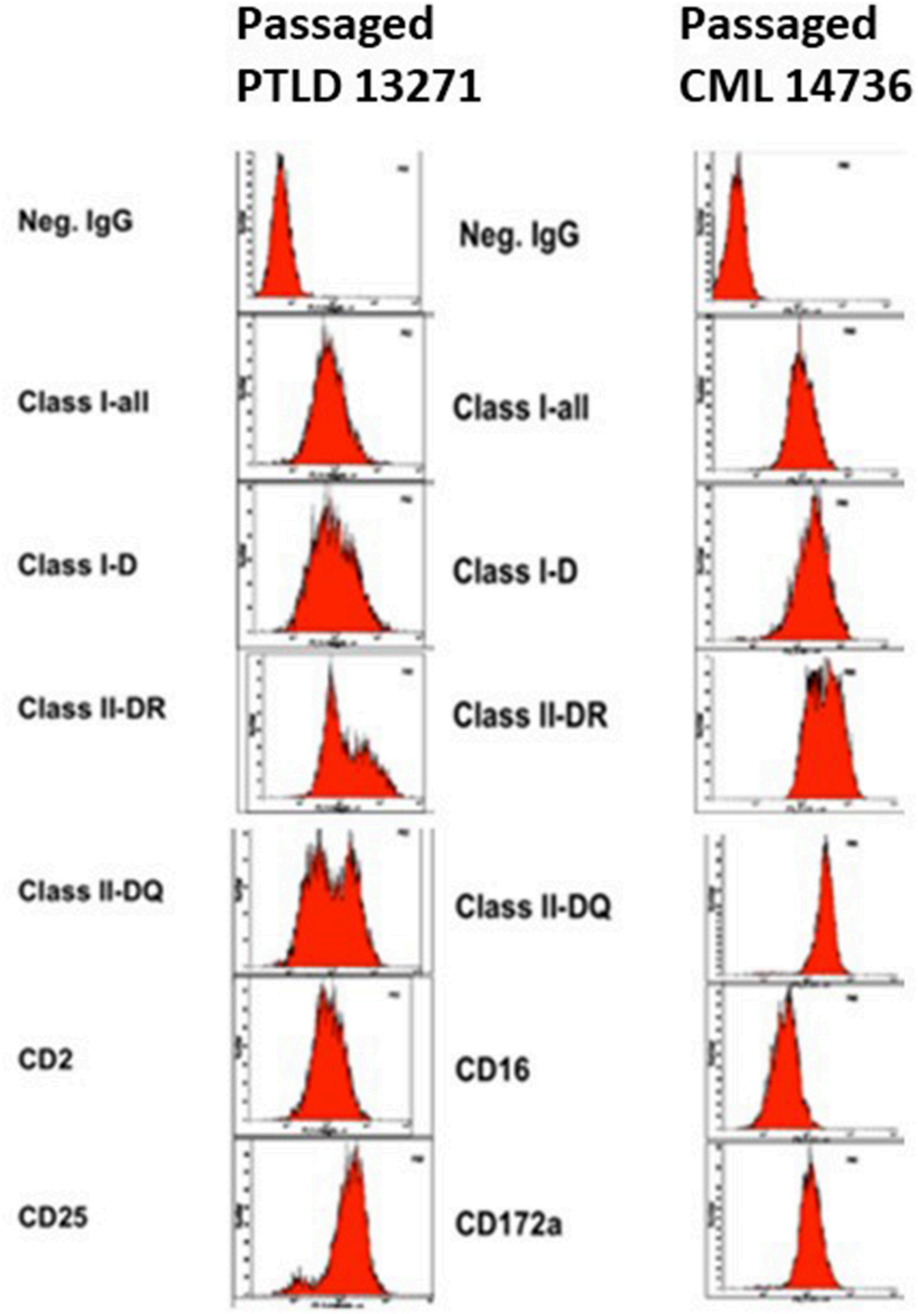
Phenotype of passaged PTLD 13271 and passaged CML 14736.

Tumors harvested from both PTLD 13271 and CML 14736 affected NSG mice were phenotyped for the presence of swine markers (Figures 2A,B). An anti-mouse MHC Class I marker, H-2Dd, was used to assess for the presence of mouse cells. All harvested tumors stained positive for swine and were negative for murine markers, demonstrating harvested tumors were of swine origin. We then compared the phenotype of the original tumor cell lines and the tumor cells harvested after *in vivo* passage. Overall, *in vivo* passaged tumor cells retained similar staining, size, and granularity. PTLD 13271 and passaged PTLD 13271 both expressed MHC class I and class II, CD2, and CD25 (Figure 3). Expression of these markers is consistent with activation (CD25) and a B-cell phenotype (CD2), in addition to lacking markers for other cell types. The original CML 14736 tumor cell line and the tumor cells recovered from NSG mice also had similar staining, size, and granularity. Both CML lines stained positive for MHC I-all, Class I-D, and Class II-DQ (Figure 3). All tumors remained negative for T-cell markers (CD3, CD4, CD8) and stained positive for the myeloid markers CD16 and CD172a (5). In summary, tumor cells harvested following *in vivo* passage in NSG mice were consistent with the original swine tumors, indicating growth of swine PTLD and CML across xenogeneic barriers.

### Re-passage of PTLD 13271 Leads to Increased Burden of Metastasis

**Figure 4:**
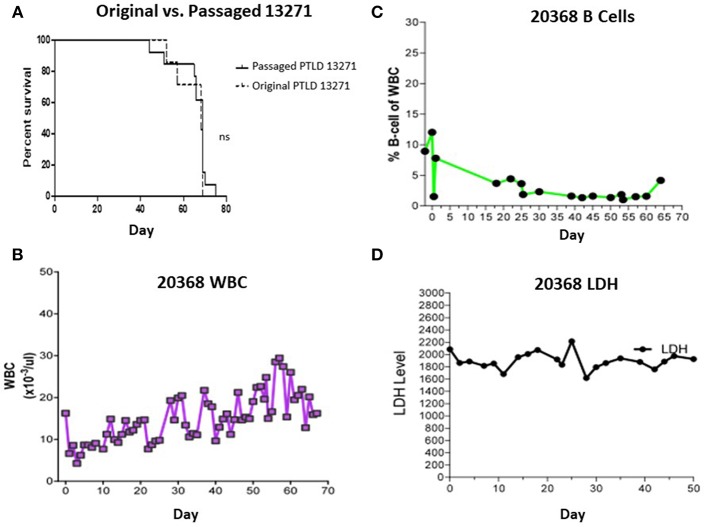
**(A)** Survival comparison between the 1st and 2nd passage of PTLD 13271 in NSG mice. **(B)** WBC count of swine 20368 throughout the course of the experiment. The WBC stayed within normal limits and did not demonstrate PTLD. **(C)** B cells as a percent of total WBC in swine 20368 as determined by FACS analysis. The levels remained within normal limits. **(D)** Serum LDH levels of swine 20368. The levels remained normal throughout the experiment.

Swine PTLD tumors isolated following passage through NSG mice were expanded *in vitro* and administered to a second cohort of mice. 10 × 10^6^ passaged 13271 tumor cells were injected into 13 NSG mice. 100% of NSG mice developed PTLD and died within 70 days (Figure 4A). Although an increased time to mortality in the second cohort of NSG mice was not observed compared to the first cohort, there was increased organ involvement with tumor in the second cohort of mice including lung, eye, ovary, stomach, and cardiac lymph nodes.

### Adoptive Transfer of PTLD 13271 Into Swine Utilizing a Tolerance Induction Regimen Does Not Result in Tumor Growth

Based on the success observed in the murine experiments, we first attempted to induce PTLD in a naïve SLA^AD^ miniature swine by adoptive transfer of original (non-passaged) PTLD 13271 tumor cells. In an attempt to improve outcomes compared to previous large animal tumor experiments, a tolerance protocol was attempted. The animal was conditioned using a non-myeloablative regimen consisting of 100 cGy TBI, T-cell depletion (using CD3 immunotoxin), and cyclosporine for 45 days. This regimen has previously led to stable mixed chimerism and immunological tolerance across MHC barriers when used as part of a haploidentical HCT protocol (7, 8). Cells were prepared and administered as described in the methods section. Swine 20368 received three doses of cells: 274 × 10^6^ cells/kg, 300 × 10^6^ cells/kg, and 437 × 10^6^ cells/kg. During the post-transplant course,CBC, flow cytometry analysis for B-cell expansion, and LDH levels were inconsistent with PTLD development (Figures 4B–D). No physical signs of tumor growth, such as lymphadenopathy, were observed. Cell mediated lympholysis (CML) assays on days 25 and 50 showed no evidence of sensitization to tumor antigen (data not shown). The animal was euthanized on day 70 and necropsy was unremarkable.

### Transduction of PTLD 13271 With a GFP Expressing Lentiviral Vector Selects for Aggressive Subclones Leading to Faster *in-vivo* Growth

**Figure 5:**
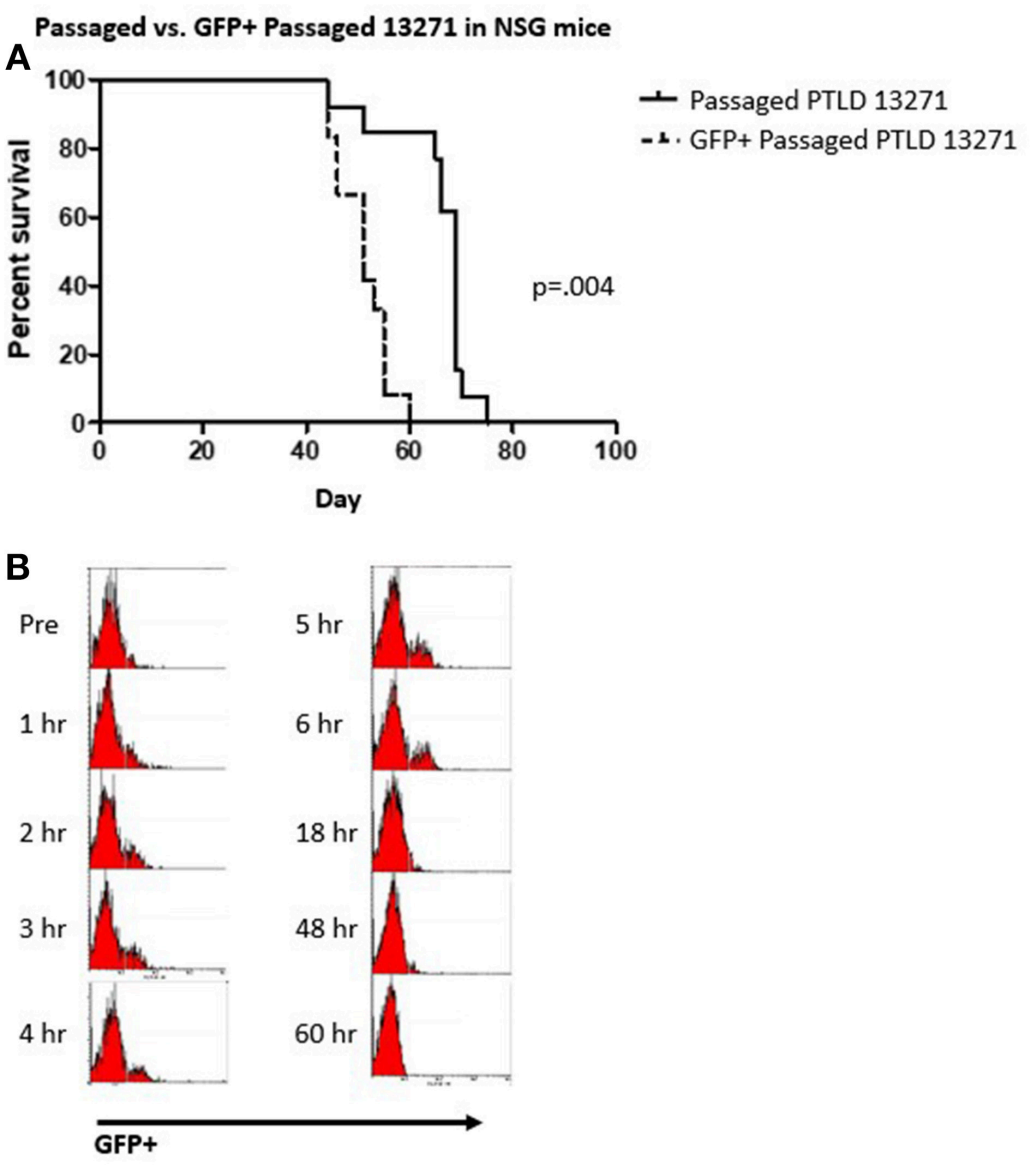
**(A)** Survival curve demonstrating the difference between the passaged PTLD 13271 and GFP+ passaged PTLD. The MST decreased from 69 to 52 days (*p* = 0.004). **(B)** Flow cytometry analysis showing the progression of GFP positive cells taken from the peripheral blood of 20984 after the injection of GFP+ passaged PTLD 13271 cells at time 0. Evidence of GFP in the PB is observed 48 h post injection.

To facilitate tracking of tumor cells *in vivo*, we transduced the *in vivo* passaged PTLD 13271 tumor cell line with a GFP expressing lentiviral vector. To determine if GFP transduction affected tumor cell kinetics *in vivo*, GFP+ and GFP– tumor cells were then sorted and transferred into a naïve cohort of NSG mice. Fifteen NSG mice were injected with 10 × 10^6^ GFP+ tumor cells and fifteen NSG mice were injected with 10 × 10^6^ GFP– tumor cells. All mice died from swine PTLD. Interestingly, mice injected with GFP+ tumor cells had a significantly decreased MST compared to those injected with GFP- tumor cells (52 vs. 69 days (*p* = 0.004) (Figure 5A).

### Adoptive Transfer of GFP+ PTLD 13271 Cells Into Swine Facilitates *in vivo* Tumor Tracking

Based on the above results, manipulation of the tumor cells through *in vivo* passage and GFP transduction created a more aggressive and easily trackable tumor. We then attempted to induce tumorigenesis in another naïve SLA^AD^ swine (20984) using a preparatory regimen consisting of 1,000 cGY thymic irradiation, T cell depletion with CD3 immunotoxin and a 60-day course of cyclosporine. We chose to substitute thymic irradiation as prior experiments in our model showed thymic irradiation to be an independent risk factor for development of PTLD (10). In the post-infusion period, GFP+ cells were observed in the peripheral blood for up to 48 h but were undetectable after that (Figure 5B). The animal was monitored for physical and laboratory signs of PTLD, including WBC and LDH levels, but no evidence of tumor growth was detected. Upon euthanasia, there was no evidence of tumor growth.

## Discussion

We report the development of an aggressive, traceable PTLD tumor cell line through *in vivo* passaging in immunocompromised mice and the *in vitro* addition of a GFP expressing lentiviral vector to facilitate *in vivo* tracking. Previous studies have shown that serial passage of tumor cells through mice may produce tumor lines that are more resistant by facilitating the loss of tumor antigens and becoming less immunogenic. Although the MST of mice injected with original and passaged tumor was similar, overall the passaged tumors were more aggressive based on increased tumor burden and metastases.

CML 14736 and PTLD 13271 swine tumor cell lines were chosen for these experiments based on previous experience with these cell lines (5). Spontaneously arising swine CML has been previously characterized in our laboratory and shown to be very similar to human CML (9). Similarly, swine PTLD has been well-described by our group and shown to correlate closely with human PTLD, including its association with a viral infection, PLHV-1 (11). After the initial passage through mice, CML 14736 grew much more slowly (MST = 190 days) than the PTLD 13271 (MST = 69 days) and had a lower engraftment rate (68 vs. 100%). Slower growth would be expected in CML, as it is a chronic leukemia (9). For these reasons, PTLD 13271 was selected for adoptive transfer studies into swine. One of the drawbacks of the PTLD 13271 cell line is that it did not originate from an inbred strain, thus harboring PLHV minor antigens and making it potentially more immunogenic than the inbred CML 14736. Future studies will evaluate the adoptive transfer of passaged CML 14736 tumor cells into swine.

A strength of this xenogeneic murine model of swine PTLD is its reproducibility. All NSG mice injected with PTLD 13271 tumor cells developed illness in a very precise time frame, allowing for definitive therapeutic endpoints. In previously published studies, we tested the therapeutic effect of two different fusion immunotoxins in this model (12, 13). Those studies evaluated the use of a swine specific CTLA-4 fusion immunotoxin (CTLA-4 FT) and a swine specific IL-2 fusion immunotoxin (IL-2 FT). Use of both immunotoxins demonstrated a prolonged MST. These studies demonstrate the utility of our model in testing novel swine anti-PTLD therapies.

We observed altered tumor cell kinetics when PTLD 13271 cells were transduced with GFP-expressing lentiviruses, resulting in decreased MST. Our transduction rate was small, 5%, but consistent with previous reports of the transduction rate of B-cells using a lentivirus with a VSV-G pseudotype (14). One possible explanation for the increased tumorigenicity of the GFP+ cells is that the insertion site of the lentivirus into the cell genome was a site that was highly expressed, therefore, selecting for highly proliferating cells by flow cytometry. Studies have shown that retrovirus and lentivirus vectors are more likely to insert near these regions than in other random locations (15). If the GFP lentivirus inserted into the most rapidly dividing cells or cells with high viral titers, this could explain why the tumor cells affected the mice more quickly than GFP-negative tumor cells.

Following initial passage of PTLD 13271 through NSG mice, harvested tumor was transferred to an MHC matched swine. Despite using an induction protocol sufficient to achieve tolerance across MHC barriers (8), tumor growth was not achieved. We then hypothesized that a more “aggressive” tumor combined with a slightly more myeloablative preparatory regimen could optimize tumor engraftment. A second swine (20984) was conditioned with 1,000 cGY of thymic irradiation instead of 200 cGY TBI and challenged with GFP+ labeled tumor cells, which had shown a decreased MST in mice after a second round of *in vivo* passage. Thymic irradiation has previously been shown to be a risk factor for development of PTLD in our model (10) where immature (and potentially reactive) thymocytes are eliminated. Tumor cells were only administered once to reduce the possibility of sensitization secondary to repeated antigen exposure. Despite these adjustments, tumor growth was not observed, including on necropsy. The reason for the lack of growth could be attributed to tumors having migrated to peripheral sites where we were unable to find them, death secondary to senescence, or perhaps requirement of more time than what was allowed in our IACUC protocol. Importantly, no sensitization was observed in our recipients who would have indicated active rejection. Further optimization of our tolerance induction approach will focus on inducing a state of mixed chimerism and immune tolerance using our established HCT protocol, followed by delayed injection of tumor cells of identical haplotype. We hypothesize that an existing state of mixed chimerism and immune tolerance will facilitate tumor engraftment.

In summary, in this study we have developed a xenogeneic murine model of swine PTLD that has since been used to test novel swine anti-PTLD therapies. Further, transduction of this aggressive PTLD cell line with GFP allowed for tracking tumor cells *in vivo* in miniatures wine. Future approaches at creating a large animal tumor model will involve further optimization of this tolerance induction strategy.

## Data Availability

All datasets generated for this study are included in the manuscript and/or the supplementary files.

## Ethics Statement

This study was carried out in accordance with the recommendations of Massachusetts General Hospital IACUC committee. The protocol was approved by the MGH IACUC committee.

## Author Contributions

RD-S, MS, and AM wrote the manuscript. MS and AM generated the figures, performed experiments, and analyzed data. IH and RH performed experiments. CH and RD-S designed the research.

### Conflict of Interest Statement

The authors declare that the research was conducted in the absence of any commercial or financial relationships that could be construed as a potential conflict of interest.

## Abbreviations

PTLD: post-transplant lymphoproliferative disorder
CML: chronic myelogenous leukemia
HCT: hematopoietic cell transplantation
NSG: NOD/SCID IL2 γ^−/−^
GFP: green fluorescent protein
MGH: Massachusetts General Hospital
MHC: major histocompatibility complex
SQ: subcutaneous
IP: intraperitoneal
IV: intravenous
SLA: swine leukocyte antigen
SRAC: Subcommittee on Research Animal Care
CyA: Cyclosporine
TBI: total body irradiation
TI: thymic irradiation
LDH: lactate dehydrogenase
MST: Mean Survival Time
CBC: Complete Blood Count
CML: Cell Mediated Lysis
PLHV: Porcine Lymphotropic Herpes Virus.

## References

[1] Borowsky AD Jose GJ Robert MJ Robert CD Comparative pathology of mouse models of human cancers. Comp Med. (2003) 53:248–58. Available online at: https://www.ingentaconnect.com/content/aalas/cm/2003/00000053/00000003/art00004?crawler=true

[2] Borowsky AD Munn RJ Galvez JJ Cardiff RD Ward JM Morse HC III. Mouse models of human cancers (part 3). Comp Med. (2004) 54:258–70. 15253271

[3] Swindle MM Smith AC Swine in the Laboratory: Surgery, Anesthesia. 2nd ed Boca Raton, FL: CRC Press; Taylor and Francis Group (2009).

[4] Sachs DH Leight G Cone J Schwarz S Stuart L Rosenberg S. Transplantation in miniature swine. I. Fixation of the major histocompatibility complex. Transplantation. (1976) 22:559–67. 137560 10.1097/00007890-197612000-00004

[5] Cho PS Lo DP Wikiel KJ Rowland HC Coburn RC McMorrow IM. Establishment of transplantable porcine tumor cell lines derived from MHC-inbred miniature swine. Blood. (2007) 110:3996–4004. 10.1182/blood-2007-02-074450 17702898 PMC2190613

[6] Racki WJ Covassin L Brehm M Pino S Ignotz R Dunn R NOD-scid IL2rγnull (NSG) mouse model of human skin transplantation and allograft rejection. Transplantation. (2010) 89:527–36. 10.1097/TP.0b013e3181c90242 20134397 PMC2901915

[7] Mezrich JD Haller GW Arn JS Houser SL Madsen JC Sachs DH. Histocompatible miniature swine: an inbred large-animal model. Transplantation. (2003) 75:904–7. 10.1097/01.TP.0000054839.43852.BF 12660524

[8] Cina RA Wikiel KJ Lee PW Cameron AM Hettiarachy S Rowland H. Stable multilineage chimerism without graft versus host disease following nonmyeloablative haploidentical hematopoietic cell transplantation. Transplantation. (2006) 81:677–85. 10.1097/01.tp.0000226061.59196.84 16794534

[9] Duran-Struuck R Cho PS Teague AG Fishman B Fishman AS Hanekamp JS. Myelogenous leukemia in adult inbred MHC-defined miniature swine: a model for human myeloid leukemias. Vet Immunol Immunopathol. (2010) 135:243–56. 10.1016/j.vetimm.2009.12.005 20079939 PMC2879595

[10] Matar AJ Patil AR Al-Musa A Hanekamp I Sachs DH Huang CA. Effect of irradiation on incidence of post-transplant lymphoproliferative disorder after hematopoietic cell transplantation in miniature Swine. Biol Blood Marrow Transplant. (2015) 21:1732–8. 10.1016/j.bbmt.2015.07.017 26210443

[11] Huang CA Fuchimoto Y Gleit ZL Ericsson T Griesemer A Scheier-Dolberg R. Posttransplantation lymphoproliferative disease in miniature swine after allogeneic hematopoietic cell transplantation: similarity to human PTLD and association with a porcine gammaherpesvirus. Blood. (2001) 97:1467–73. 10.1182/blood.V97.5.1467 11222395

[12] Peraino JS Schenk M Li G Zhang H Farkash EA Sachs DH. Development of a diphtheria toxin-based recombinant porcine IL-2 fusion toxin for depleting porcine CD25+ cells. J Immunol Methods. (2013) 398:33–3. 10.1016/j.jim.2013.09.006 24055128 PMC3840057

[13] Peraino JS Schenk M Zhang H Li G Hermanrud CE Neville DM Jr. A truncated diphtheria toxin based recombinant porcine CTLA-4 fusion toxin. J Immunol Methods. (2013) 391:103–11. 10.1016/j.jim.2013.02.015 23470981 PMC3688055

[14] Janssens W Chuah MK Naldini L Follenzi A Collen D Saint-Remy JM. Efficiency of onco-retroviral and lentiviral gene transfer into primary mouse and human B-lymphocytes is pseudotype dependent. Hum Gene Ther. (2003) 14:263–76. 10.1089/10430340360535814 12639306

[15] Beard BC Dickerson D Beebe K Gooch C Fletcher J Okbinoglu T. Comparison of HIV-derived lentiviral and MLV-based gammaretroviral vector integration sites in primate repopulating cells. Mol Ther. (2007) 15:1356–65. 10.1038/sj.mt.6300159 17440443

